# Reduced hTERT protein levels are associated with DNA aneuploidy in the colonic mucosa of patients suffering from longstanding ulcerative colitis

**DOI:** 10.3892/ijmm.2014.1708

**Published:** 2014-03-20

**Authors:** MARIANN FRIIS-OTTESSEN, PAULA M. DE ANGELIS, AASA R. SCHJØLBERG, SOLVEIG N. ANDERSEN, OLE PETTER F. CLAUSEN

**Affiliations:** 1 Division of Diagnostics and Intervention, Department of Pathology, Oslo University Hospital, Rikshospitalet, 0424 Oslo, Norway; 2 Department of Pathology, University of Oslo, 0424 Oslo, Norway; 3 Department of Pathology, Akershus University Hospital, Division of Medicine and Laboratory Sciences, University of Oslo, 1474 Nordbyhagen, Norway

**Keywords:** aneuploidy, dysplasia, immunohistochemistry, telomerase, ulcerative colitis

## Abstract

Longstanding ulcerative colitis (UC) is a disease of chronic inflammation of the colon. It is associated with the development of colorectal cancer through a multistep process including increasing degrees of dysplasia and DNA-ploidy changes. However, not all UC patients will develop these characteristics even during lifelong disease, and patients may therefore be divided into progressors who develop dysplasia or cancer, and non-progressors who do not exhibit such changes. In the present study, the amount of hTERT, the catalytic subunit of the enzyme telomerase, was estimated by using peroxidase immunohistochemistry (IHC) in a set of progressor and non-progressor UC colectomies. The protein levels in the colonic mucosa of the progressors and non-progressors were compared, and further comparisons between different categories of dysplastic development and to DNA-ploidy status within the progressors were made. Levels of hTERT were elevated in the colonic mucosa of the progressors and non-progressors when compared to non-UC control samples, but no difference was observed between the hTERT levels in the mucosa of progressors and non-progressors. The levels of hTERT associated with levels of Ki67 to a significant degree within the non-progressors. hTERT expression in lesions with DNA-aneuploidy were decreased as compared to diploid lesions, when stratified for different classes of colonic morphology. Our results indicate an association between hTERT protein expression and aneuploidy in UC-progressor colons, and also a possible protective mechanism in the association between hTERT and Ki67, against development of malignant features within the mucosa of a UC-colon.

## Introduction

Ulcerative colitis (UC) is an inflammatory bowel disease that is associated with an elevated risk of developing colorectal cancer, and it is estimated that 10% of patients suffering from UC for >10 years will develop colorectal cancer (1,2). The underlying pathogenesis is not fully known, but chronic inflammation distorts mucosal morphology and induces dysplasia and subsequently cancer. Carcinomas occur mainly after long-term illness of 8–10 years (3), and develop through low- and high-degree dysplasia. It is reported that patients presenting only one lesion of low-degree dysplasia may also harbour carcinomas (4). The colonic mucosa of UC patients may also harbour severe molecular abnormalities, such as chromosomal instability (5) and DNA aneuploidy. DNA aneuploidy may be present in dysplastic and non-dysplastic mucosa of UC patients, and is reported to be connected with the duration of disease (6–9). It can be characterized as an independent risk factor for the development of adenocarcinoma in UC (10,11). UC-patients who develop dysplasia or adenocarcinomas are usually considered progressors, whereas patients who do not develop these phenotypes during a lifetime of UC are considered non-progressors. Since DNA-aneuploidy can be considered an independent risk factor for malignancies in UC-colon mucosa, we have also included this as a defining characteristic of a UC-progressor case. The mechanisms behind what makes a UC-colon a progressor or a non-progressor are not fully known, and it is therefore of interest to examine the differences in molecular features of the mucosa between these two types of UC-affected colons. Molecular characteristics of dysplastic as well as non-dysplastic lesions within a progressor that are not found in non-progressors, could be a contribution to the understanding of mechanisms behind carcinogenesis in UC colons, which could serve as a marker for individuals at risk.

Telomerase is a ribonucleoprotein capable of extending the telomeric sequence, generally known to be active in germline cells and inactive in most somatic cells. The two main subunits of telomerase are the catalytic subunit TERT-telomerase reverse transcriptase (hTERT in humans), and TR (TER), the RNA component (hTR or hTER in humans). In addition to hTERT and hTR, a range of accessory proteins are also closely associated with the complex (12). The level of hTERT is generally assumed to be a limiting factor for assembly of the telomerase complex, and it is reported that hTERT may also play a role in cell proliferation, separate from its role in telomere elongation (13). Telomerase activity is frequently reported in cancer cells, and ~80–90% of all solid tumours, including colorectal cancer, have reported telomerase activity (14–16). Telomerase enables cancerous cells to achieve replicative immortality, which is one of the hallmarks of cancer (17). Telomerase activity may therefore increase the lifespan of a cell, resulting in an accumulation of genetic alterations in the cell that again may contribute to the development of cancer (18). In UC mucosa, however, reports on telomerase activity vary from reduced levels (19), levels not differing from non-UC colons (20–22), to reports on elevated activity (23,24). All these investigations used versions of the Telomeric Repeat Amplification Protocol (TRAP)-assay or PCR-ELISA, valid methods for measuring telomerase activity in a sample by using tissue extracts. Due to the often high levels of inflammation in a UC colon, elevated levels of macrophages and neutrophils are present, and tissue extracts from the colonic mucosa of UC patients may therefore comprise these cell types. A TRAP-assay from colonic mucosal cells may therefore not differentiate between telomerase activity in macrophages and leucocytes in the tissue from that of the epithelial cells, making results difficult to interpret. Notably, in a study on telomerase activity in UC colonic mucosa, where mucosal cells had been separated from stromal cells the results showed different levels of activity in the two sets. Levels of telomerase activity were reported as low in dysplastic mucosa, and a correlation between telomerase activity and inflammation was detected. In this report, DNA-status was not included (22). It has also been speculated as to whether elevated levels of telomerase-activity in UC mucosa are a direct result of enhanced cell proliferation in actively inflamed colon tissue (24).

In the present study, we used immunohistochemistry (IHC) to assess hTERT levels in UC material as it provides the advantage of assessing the protein expression in specific cell types within the tissue examined, thus allowing for the exclusion of macrophages and neutrophils that would obfuscate hTERT level data. We assessed hTERT protein levels using IHC in the colonic mucosal cells of a set of progressor and non-progressor colons of patients suffering from longstanding UC, to investigate whether any differences in hTERT expression were related to progressor status, mucosal dysplastic development or to DNA-ploidy status.

## Materials and methods

### Patients

Thirty patients suffering from longstanding UC were included in this report. All the patients had suffered from UC for >10 years prior to colectomy, and some patients had suffered as long as 30 years. Patients also varied widely in age at the time when symptoms first presented (from 10 to 60 years old). The 10 non-progressor patients included 5 males and 5 females. The progressors included 17 males and 3 females. Use of this material for research purposes received ethical approval from the Regional Ethics Committee, REK S-06062.

### UC colectomies: Progressors and non-progressors

The colectomy specimens have previously been described by Burum-Auensen *et al* (25). The colectomies (n=30) were grouped into progressors and non-progressors, revealing 10 non-progressors that presented no dysplastic lesions, and 20 progressors that all presented at least one area of dysplasia/cancer. The majority of cases also presented DNA aneuploidy.

At least eight sites from each colectomy were examined, and within the progressors 83 non-dysplastic areas were identified, 31 areas indefinite for dysplasia, 29 areas with dysplasia and 8 adenocarcinomas. Since our analyses focused on precancerous morphology changes, the adenocarcinomas were excluded. A total of 18 non-dysplastic and 7 dysplastic areas revealed DNA aneuploidy. The progressor lesions are shown in Table I. By definition the non-progressor lesions were diploid and non-dysplastic.

### hTERT IHC

**Figure 1:**
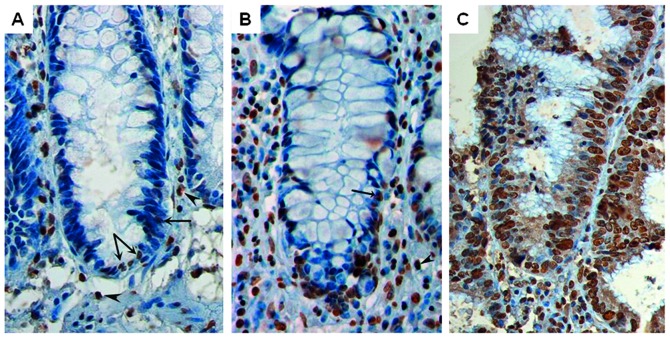
Immunohistochemistry (IHC) for hTERT. (A) Non-UC control sample (from full section), (B) ulcerative colitis (UC)-lesion with low hTERT levels and (C) UC-lesion with high hTERT levels. Images of UC colons are from tissue microarray (TMA)-cores. Arrows mark colonic mucosal cells positive for hTERT in low expression levels, arrowheads mark hTERT-stained leucocytes. Images are ×400 magnification.

**Figure 2:**
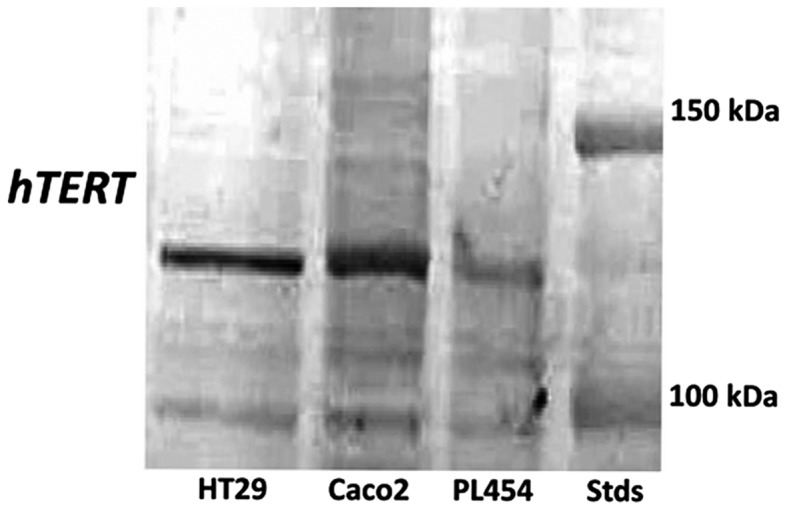
Western blot analysis confirming the specificity of ab5181, a monoclonal antibody for hTERT expression. The antibody was specific, showing a single band at 127 kDa when tested using several cancer cell lines.

Tissue microarrays (TMAs) from eight sites within each colon were made using a Beecher tissue microarrayer as described previously (25). Core size was 0.6 mm. All cores were previously evaluated by an experienced pathologist (OPFC). At least two tissue cores from each mucosal region were sampled. Two tonsillar sections were used as positive controls. Sections (4 μm) were exposed to 0.5% H_2_O_2_ solution for 10 min, followed by antigen retrieval in the citrate buffer at pH 6.0. Incubation of TMAs with the primary antibody against telomerase [mouse monoclonal ab5181, dilution (1:500); Abcam, Cambridge, UK], was performed for 1 h at room temperature. Staining was performed using a Ventana Nexes machine using Ventana Iview DAB detection kit (Ventana Medical Systems, Tucson, AZ, USA) according to the manufacturer’s instructions. Sections stained with Tris-buffered saline (TBS) instead of primary antibody served as negative staining controls. hTERT protein expression was defined for each sample as the percentage of positive cells out of 1,200 randomly selected mucosal epithelial cells. Only cells with nuclear staining were counted as positive for hTERT-expression. hTERT-staining of a non-UC control sample and progressor lesions with high and low hTERT levels are presented in Fig. 1. The antibody was tested for specificity using several human cancer cell lines, and a single band of 127 kDa was detected (Fig. 2).

We have previously presented the immunostaining for Ki67 for this material, showing significantly elevated levels of Ki67 in UC colons compared to non-UC controls (25).

### Statistical analysis

As each patient included in this study contributed with more than one biopsy, we evaluated the levels of protein expression in relation to the morphologic parameters, as well as the association analyses of protein levels for hTERT and Ki67, by using a multilevel model that compensates for patient differences. The linear mixed model (LMM), with restricted maximum likelihood (REML) estimations and a Bonferroni post-hoc test was performed. Tests were performed in PASW^®^ statistics 18 (Chicago, IL, USA). All tests were two-sided and a p-level of 0.05 denoted significance.

## Results

### TMA evaluation

TMAs do not consistently exhibit full colonic crypts as observed in whole sections, but since we found the expression of hTERT in our study to be evenly distributed throughout the colonic mucosa we concluded that hTERT protein levels could be estimated reliably (data not shown).

As Ki67 protein expression is linked to the growth fraction in UC-colonic mucosa we did not consider TMAs as reliable in assessing Ki67 expression related to dysplastic development.

The assessment of Ki67 protein expression was performed within the same tissue cores as for hTERT protein assessments, thus evaluation of the association between hTERT and Ki67 was considered to be reliable.

### Levels of hTERT in the colonic mucosa of progressors vs. non-progressors

**Figure 3:**
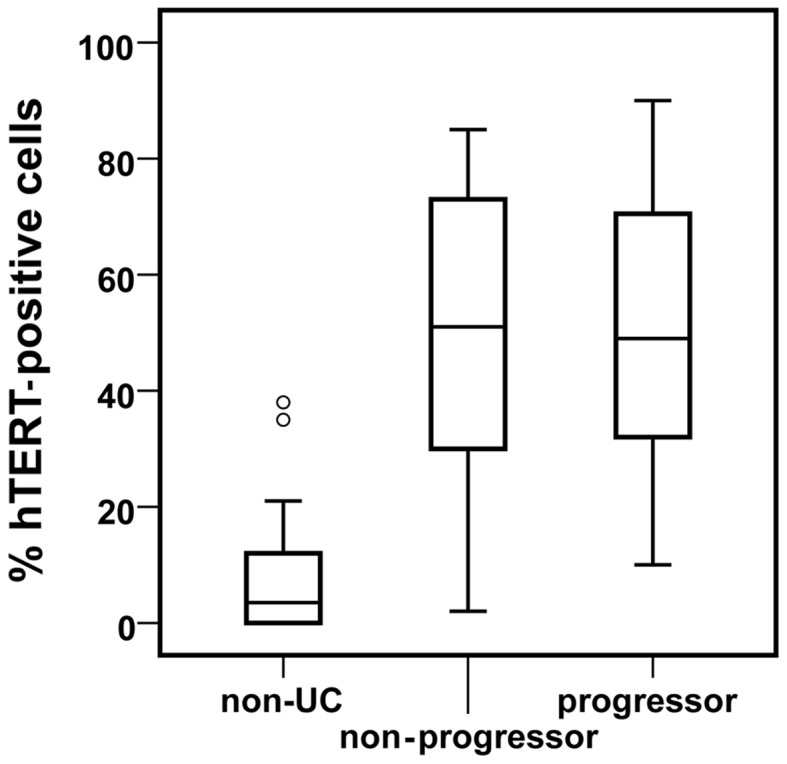
hTERT in ulcerative colitis (UC) progressors, non-progressors and non-UC controls. Protein levels of hTERT detected by immunohistochemistry (IHC) in progressors, non-progressors and non-UC controls.

Levels of hTERT were significantly elevated (p<0.001) in the colonic mucosa of progressors and non-progressors, compared to non-UC controls (Fig. 3). No difference was observed comparing progressor and non-progressor colectomies. Statistically elevated levels of Ki67 in overall UC colons compared to non-UC controls have been previously presented (25).

### Levels of hTERT within the colonic mucosa of progressor colectomies

The progressors were divided according to age at onset, as it has been recently shown that progressors with late onset of UC (> 50 years old) differed in telomere biology from progressors with early onset of UC (<50 years old) (26). The results yielded no statistical difference in the protein levels of hTERT when comparing late and early onset UC (p=0.2).

**Figure 4:**
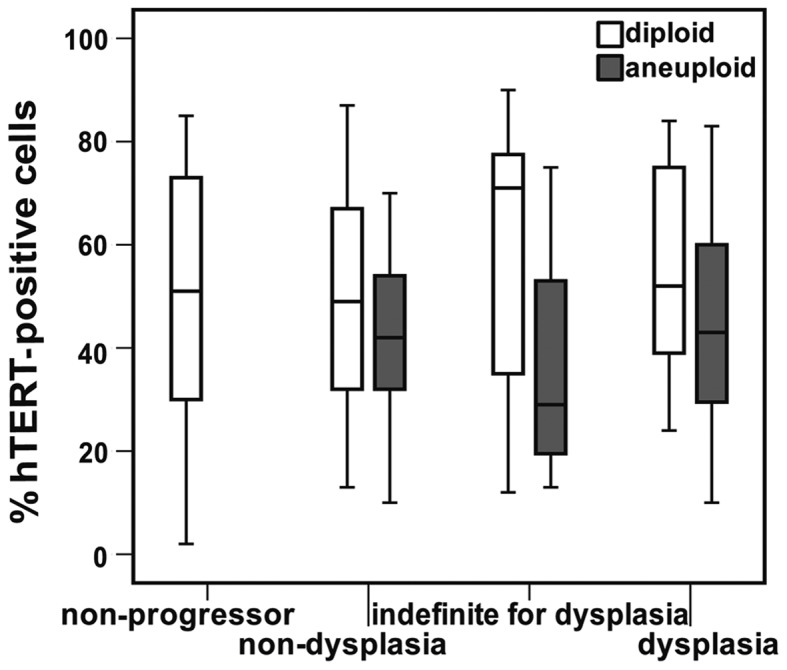
hTERT in diploid and aneuploid lesions of progressors. Expression of hTERT in non-progressors and within areas with different morphologies harbouring diploid or aneuploid populations in progressors.

No statistically significant difference in the levels of hTERT expression was detected between diploid lesions and lesions presenting aneuploidy, without correcting for differences in mucosal morphology (p=0.12). No significant differences in hTERT levels were identified between non-dysplastic lesions, lesions indefinite for dysplasia and dysplastic lesions without correcting for DNA-ploidy status (p=0.14). However, when stratifying for mucosal morphology and comparing hTERT protein levels within diploid lesions with those harbouring aneuploid populations, we found that the aneuploid lesions tended to have less hTERT expression than the diploid counterparts (Fig. 4).

Within the non-dysplastic aneuploid and diploid lesions of the progressor colons the hTERT levels differed to a statistically significant extent (p=0.037) when using LMM accounting for the differences between the patients. The p-values detected using LMM for DNA-ploidy status within each morphologic group are presented in Table II. By ignoring patient differences, and using a t-test, a statistically significant difference was found between hTERT levels stratified for DNA-ploidy status within the lesions scored as indefinite for dysplasia. Diploid lesions had higher levels of hTERT than aneuploid lesions.

### Associations between protein levels of hTERT and Ki67 in the colonic mucosa of progressors and non-progressors

An association analysis between hTERT and Ki67 revealed statistically significant results within the non-progressors (p=0.047) when using LMM, compensating for patient variation. No association was detected within the different lesions of the progressors (Table III). No association was detected between the protein levels of hTERT and Ki67 within the progressors when stratifying for DNA-ploidy status.

All analyses were also performed excluding all cases harbouring adenocarcinomas. This did not alter the results to any statistically significant degree.

## Discussion

In the present study, we found significantly raised levels of hTERT protein in the mucosa of both progressor and non-progressor UC colectomies compared to non-UC control samples (p<0.001), but no significant difference was detected between hTERT levels in the progressor and non-progressor colectomies. UC is reportedly a disease of accelerated aging of colonic mucosa (27), with an elevated cell division rate as documented by Greco *et al* (28). The fact that we detected similar hTERT levels in progressor and non-progressor colectomies is consistent with those studies, as the patients had suffered from UC for >10 years. Elevated levels of hTERT were found in mildly active UC in the mucosa of patients suffering from UC on average 6 years (29).

For examination of possible differences in the levels of hTERT within the progressors we stratified the areas of the 20 progressor colectomies by morphological characteristics, and compared diploid areas with areas containing aneuploid clones, using LMM. This comparison showed a pattern of lower hTERT expression in aneuploid lesions within areas of similar morphology. Within the non-dysplastic lesions this difference was statistically significant (p=0.037). If each lesion was included in the analysis as independent data entries (Student’s t-test), we found a significant difference between hTERT levels in diploid and aneuploid lesions indefinite for dysplasia. However, the protein levels of hTERT vary between patients, a fact that potentially affects our statistical findings, creating false positives. Several of the aneuploid lesions of indefinite dysplastic morphology were found within the same colon (Table I), and this could skew our results. We therefore found the p-values yielded by the LMM analysis controlling for patient variations to be valid. The hTERT-protein expression in diploid, non-dysplastic lesions did not differ from the levels found in the non-progressors, which are all non-dysplastic and diploid. This shows that when the confounding factor of differences in mucosal morphology is accounted for, reduced levels of hTERT are linked to DNA aneuploidy and possibly also associated with its development. Increased levels of hTERT may enhance the proliferative activity of the inflamed tissue harbouring increased levels of reactive oxygen species (ROS), and possibly contribute to the development of dysplasia and cancer. It has been demonstrated that UC colons have enhanced cell proliferation (24) and elevated levels of ROS (30). Both these agents are reported to facilitate telomere shortening. Too short or even missing telomeres can induce breakage-fusion-bridge (BFB) cycles, which again can lead to chromosomal instability (5) and DNA aneuploidy (31,32). Elevated levels of BFB were shown in UC-progressor colons, but DNA-ploidy status of the lesions examined was not investigated (31). Activation of telomerase can prevent BFB-cycles by adding telomeric sequences to short telomeres or broken chromosome ends (33). Our results showing less hTERT present in lesions that contain aneuploid cell populations are consistent with these results.

UC progressors have been shown to differ in mean telomere length depending on the patients’ age at disease onset, where early onset (<50 years of age when diagnosed) harboured shorter telomeres than those observed in UC progressors with later UC onset (26). All our patients had suffered from active colitis for >10 years at the time of colectomy and all had presented extensive colitis. Only two progressors were diagnosed with UC after the age of 50, and these did not differ in hTERT expression from the patients diagnosed at an earlier age.

It is possible that any differences in hTERT levels of the colonic mucosa between the progressors and non-progressors were levelled out by continuous impairment of the colonic mucosa due to inflammation and regeneration. Telomeres in UC colonic mucosa are reported to shorten more rapidly than in non-UC mucosa (27,31), and activation of telomerase might be a response to this attrition. This could indicate that hTERT expression is not a biomarker for differentiating a progressor colon from a non-progressor colon prior to colectomy.

A study of four colectomies from patients suffering from UC for >20 years revealed a regional correlation between dysplasia and telomerase activity measured by a version of the ELISA. One patient did not present dysplasia or telomerase activity (23). However, the ELISA method used in the study detected assembled telomerase enzyme complexes, and it was suggested that lack of detected telomerase activity in some of the samples could be due to degradation of the RNA component of the holoenzyme prior to sampling (23). IHC of hTERT may omit tissue-based problems such as partial degradation, as it is based on visual examination of stained formalin or alcohol-fixed, paraffin-embedded tissue.

IHC facilitates the investigation of protein expression in specific cell types within a tissue. This feature can prove valuable when examining UC colons, where a high percentage of leucocytes are generally present in the mucosa. Examining hTERT protein expression by IHC allowed us to assess the differences in the extent of hTERT expression in the colonic mucosal cells, without the confounding contributions from mucosal leukocytes. In a report examining coronary plaques, neutrophils were found to have elevated levels of telomerase activity (34). This is confirmed in our study by the presence of hTERT-positive leucocytes in the lamina propria (Fig. 1).

However, immunohistochemical detection of hTERT has proven to be a difficult task, as some antibodies can also bind to other proteins not associated with telomerase activity (35), antibodies that are not commercially available, or those that are commercially available but have not been proven to be specific (i.e., non-specific cytoplasmic rather than specific nuclear staining). As new antibodies binding to hTERT have become available and tested for binding specificity, reports of hTERT-expression have emerged. Elevated levels of hTERT in precancerous lesions have been identified in gastric tissue (36), and colonic adenocarcinomas (37). The hTERT protein levels of colonic mucosa may provide insight into the transition from normal-looking mucosal morphology towards a possible colorectal cancer, as normal colonic mucosa has low hTERT levels, whereas colorectal cancers have high levels of hTERT (38). In our study the nuclear hTERT staining was very distinct. The monoclonal hTERT antibody used was specific, as confirmed by western blotting of several human cancer cell lines that showed a single band at 127 kDa as expected (Fig. 2). We have previously shown, that progressors harboured significantly more ultra-short telomeres compared to non-progressor colons, and that the difference remained statistically significant when we compared the diploid, non-dysplastic progressor lesions to the non-progressors. In terms of mean telomere length, no difference was found between progressors and non-progressors (39). Thus, an association between mean telomere length and hTERT protein levels seems to exist, whereas no association was observed between the amount of ultra-short telomeres and levels of hTERT in longstanding UC.

In a previous study, our group showed that the proliferation marker Ki67 was significantly elevated in UC colons compared to non-UC control samples, thus confirming that proliferation is enhanced in UC colonic mucosa (25). Also, protein expression of Ki67 has been shown to increase with advancing degree of growth fraction due to the developing stage of dysplasia in the colonic mucosa of the UC colon (40). We found that hTERT expression was significantly associated with the expression of Ki67 within the non-progressor lesions. Within the progressors this association was lost, even when diploid, non-dysplastic lesions were examined separately. Together with our findings of a borderline significant p-value for association between hTERT and Ki67 within progressor non-dysplasia, and no significance detected within the increasing levels of distorted morphology (Table III), it seems the association between proliferation and hTERT protein expression is lost during the development of dysplasia. The lack of difference in hTERT protein levels between progressors and non-progressors, together with elevated amounts of ultra-short telomeres identified in the progressor lesions and the hTERT/Ki67association found only within non-progressors leads to the hypothesis that the positive association between hTERT and Ki67 in the non-progressors may be a protective agent against shortening of the cells telomeres.

In conclusion, we have shown that the protein levels of hTERT were significantly elevated in the mucosa of progressors and non-progressor UC colons compared to non-UC control samples. In the progressor colons, aneuploid non-dysplastic lesions had a significantly lower expression of hTERT than the diploid non-dysplastic lesions, and diploid, non-dysplastic lesions did not differ from the non-progressors with regard to expression of hTERT protein in the colonic mucosal cells, thus low levels of hTERT associated with aneuploidy. We also found that within the non-progressors there was an association of hTERT expression and expression of the proliferation marker Ki67. No association of hTERT/Ki67 protein expression was detected in the progressors, even when only diploid non-dysplastic lesions were examined, indicating that the association of the two proteins may act as a protective mechanism against the development of progressor characteristics within a UC colon.

## Figures and Tables

**Table I:** Summary of lesions in the progressor colectomies (n=20) according to morphology and DNA-ploidy status.

		Colon specimen #																			
		
		30	70	71	99	132	159	164	169	174	176	177	191	192	199	205	225	1514	1701	1729	1789
Diploid	Non-dysplasia	5	5	2	1	1	3	1	2	7	2	3	5	4	3	6	5	6	3	0	1
	Indefinite dysplasia	0	0	0	0	2	1	2	1	0	2	1	0	1	5	2	1	1	2	2	0
	Dysplasia	0	2	0	3	1	1	2	0	1	0	0	0	1	0	1	2	0	2	6	0
	Adenocarcinoma^a^	0	0	0	0	0	0	0	0	1	0	0	0	0	0	0	2	0	0	0	0
Aneuploid	Non-dysplasia	1	0	0	0	0	3	1	1	1	4	2	2	0	0	0	0	1	1	0	1
	Indefinite dysplasia	0	0	0	0	2	0	1	0	0	0	0	0	1	1	0	0	0	0	0	3
	Dysplasia	0	0	0	1	1	1	2	1	0	0	0	1	0	0	0	0	0	0	0	0
	Adenocarcinoma^a^	1	0	1	0	0	0	0	2	0	0	0	0	0	0	0	0	0	0	0	1

a Adenocarcinomas were removed from the analyses.

**Table II:** LMM test p-values for hTERT protein levels stratified for DNA-ploidy status within different morphologic stages from progressors.

Morphology	p-value
Non-dysplasia	0.037
Indefinite for dysplasia	0.374
Dysplasia	0.565

LMM, linear mixed model.

**Table III:** P-values generated from LMM analyses for the association between hTERT and Ki67 protein expression in UC-morphology.

Morphology	p-value
Non-progressor	0.047
Non-dysplasia	0.097
Indefinite for dysplasia	0.102
Dysplasia	0.731

LMM, linear mixed model; UC, ulcerative colitis.
